# 2398. Short and Long-Term Outcomes of Patients Admitted with Infective Endocarditis Who Undergo Patient-Directed Discharge

**DOI:** 10.1093/ofid/ofad500.2018

**Published:** 2023-11-27

**Authors:** Kyle Crooker, YuTing He, Tess Hickey, Max HoddWells, Ashwini Sarathy, Torrance Teng, Sean Muniz, Jennifer Lor, Amy Chang, Bradley Tompkins, A N D R E W HALE

**Affiliations:** University of Vermont Medical Center, Burlington, Vermont; Northeast Medical Services, San Francisco, California; Larner College of Medicine at the University of Vermont, Burlington, Vermont; Larner College of Medicine at the University of Vermont, Burlington, Vermont; Larner College of Medicine at the University of Vermont, Burlington, Vermont; Larner College of Medicine at the University of Vermont, Burlington, Vermont; Larner College of Medicine at the University of Vermont, Burlington, Vermont; Larner College of Medicine at the University of Vermont, Burlington, Vermont; Larner College of Medicine at the University of Vermont, Burlington, Vermont; University of Vermont Medical Center, Burlington, Vermont; University of Vermont, Burlington, Vermont

## Abstract

**Background:**

Infective endocarditis (IE) results in high morbidity and mortality. Guidelines for IE recommend prolonged intravenous antibiotics that can result in extended hospitalization, but some patients experience patient-directed discharges (PDD) prior to completion. There is a current lack of literature clearly defining long-term outcomes and optimal treatment strategies in patients with IE who experience PDD.

**Methods:**

This is a retrospective cohort study comparing outcomes of adult patients with infective endocarditis at a single center from 2010 to 2020 who experienced patient-directed discharge versus those who did not. The primary outcomes were 30 day, 90 day, and 2 year re-admission and mortality rates related to IE. The secondary outcome was worsened IE, which encompassed pertinent clinical and microbiologic outcomes.

**Results:**

391 patients were identified and included. Of these 47 (12%) experienced PDD and 344 (88%) did not. The PDD cohort was younger (mean age 33.3y (IQR 27.7 to 37.6) vs 60.1y (IQR 37.4 to 72.5 (p< 0.01)); more likely to be on Medicaid (75% vs 29% (p< 0.01)), undomiciled (17% vs 6.4% (p=0.01)), using intravenous drugs (87% vs 27% (p< 0.01)), and have psychiatric comorbidities (53% vs 33% (p< 0.01)). The PDD cohort received a mean of 17.3 days of IV antibiotics vs 36.5 days in the controls (p< 0.01) with 26 (55%) of the PDD cohort getting oral antibiotics upon discharge with an average duration of oral antibiotics of 29.3 days. Compliance on oral antibiotics was low in the PDD cohort (46%). There was no significant difference in death related to IE (0% vs 4% (p=0.3)). 30 and 90 day readmission rates were significantly higher in the PDD cohort (38% and 47% vs 11% and 15% (p< 0.01)). The PDD cohort was significantly more likely to be readmitted with new heart valves involved (21% vs 3% (p< 0.01)), new paravalvular abscess (9% vs 1% (p< 0.01)), different causative organisms (40% vs 18% (p< 0.01)), and/or new metastatic sites of infection.

**Conclusion:**

Adult patients with IE who experience PDD are more likely to be younger, undomiciled, to use intravenous drugs and to have a psychiatric comorbidity. These patients have significantly worse outcomes from IE. Further treatment and outreach strategies in this patient population are essential to improve outcomes.

**Disclosures:**

**All Authors**: No reported disclosures

